# Assessment of Cardiac Sarcoidosis with Advanced Imaging Modalities

**DOI:** 10.1155/2014/897956

**Published:** 2014-08-28

**Authors:** Makoto Orii, Toshio Imanishi, Takashi Akasaka

**Affiliations:** Department of Cardiovascular Medicine, Wakayama Medical University, 811-1 Kimiidera, Wakayama 641-8510, Japan

## Abstract

Sarcoidosis is a chronic systemic disease of unknown etiology that is characterized by the presence of noncaseating epithelioid granulomas, usually in multiple organs. Several studies have shown that sarcoidosis might be the result of an exaggerated granulomatous reaction after exposure to unidentified antigens in genetically susceptible individuals. Cardiac involvement may occur and lead to an adverse outcome: the heart mechanics will be affected and that causes ventricular failure, and the cardiac electrical system will be disrupted and lead to third degree atrioventricular block, malignant ventricular tachycardia, and sudden cardiac death. Thus, early diagnosis and treatment of this potentially devastating disease is critically important. However, sensitive and accurate imaging modalities have not been established. Recent studies have demonstrated the promising potential of cardiac magnetic resonance imaging (MRI) and ^18^F-fluoro-2-deoxyglucose positron emission tomography (^18^F-FDG PET) in the diagnosis and assessment of cardiac sarcoidosis (CS). In this review, we discuss the epidemiology, etiology, histological findings, and clinical features of sarcoidosis. We also introduce advanced imaging including ^18^F-FDG PET and cardiac MRI as more reliable diagnostic modalities for CS.

## 1. Introduction

Sarcoidosis was first reported more than 120 years ago. It is a granulomatous inflammatory disease with an unclear etiology that affects multiple organs including the lungs, heart, skin, central nervous system, and eyes [1]. Although it is not commonly fatal, cardiac involvement may be responsible for more than two-thirds of deaths [2]. The clinical diagnosis of cardiac sarcoidosis (CS) is therefore critically important to the timely planning of therapeutic strategies.

Despite extensive research, the etiology of sarcoidosis has not been elucidated, although most evidence points to an aberrant immune response as the pathogenetic mechanism, which is driven by an unidentified antigen in genetically susceptible individuals. Although multiple candidate etiologic agents including microbial organisms and environmental agents have been identified, the results are inconclusive thus far.

Once the diagnosis of sarcoidosis is made, management may range from observation to long-term administration of steroids (often at high doses) or other immunosuppressive therapies, depending on disease severity and organ involvement [3]. Although immunosuppressive therapy may be required for CS, reliable biomarkers and effective imaging modalities have not been established for it. The diagnosis of CS is further hindered by the lack of any reliable and specific diagnostic test, as there are no imaging findings that allow for a definitive diagnosis of CS.

## 2. Epidemiology

Sarcoidosis is a systemic disease with a prevalence of about 4.7–64 in 100 000 and an incidence of 1.0–35.5 in 100 000 per year. The highest rates are reported in northern European and African-American individuals, particularly in women, and the lower rate is reported in Japan [4, 5]. Differences in prevalence and incidence are linked to age, sex, ethnic origin, and geographical location. The disease can occur in both sexes, with a female to male ratio of 1 : 1.46. Seventy percent of patients are aged 25–45 years, although a second incidence peak occurs in women older than 50 years in Europe and Japan [4, 5]. The clinical expression of sarcoidosis is affected by epidemiological and socioeconomic factors. Elderly-onset sarcoidosis is much more common in women and shows higher rates of change in general health and extrapulmonary manifestations [6]. Sarcoidosis is usually sporadic, but is familial in 3.6–9.6% of cases [7]. Siblings have a higher risk of sarcoidosis than do parents, suggesting a recessive made of inheritance with incomplete penetrance [7]. An 80-fold increase in risk in monozygotic twins lends support to the notion that genetic factors might account for two-thirds of disease susceptibility [8].

## 3. Etiology

The exact etiology of sarcoidosis remains unknown. Many studies suggest that genetic susceptibility and environmental factors contribute to disease development [9–11]. Immunologically, sarcoidosis is an exaggerated immune response to as yet unknown antigens. Data on the clinical heterogeneity of sarcoidosis strongly suggest that the pathogen-associated molecular patterns of microbial antigens can trigger or amplify inflammation. There is no evidence that sarcoidosis is an infectious disease; rather, it is likely to be an exaggerated immune response to the pathogen-associated molecular patterns of killed and partly degraded mycobacteria and propionibacteria. Other organic and inorganic substances might also trigger sarcoidosis [12]. Mycobacteria and propionibacteria persist in macrophage phagosomes because the high lipid contents in their membranes make them acid-fast, and many of their glycolipoproteins are not very soluble and resist degradation.

## 4. Histologic Findings in Sarcoidosis (Granuloma Formation)

**Figure 1:**
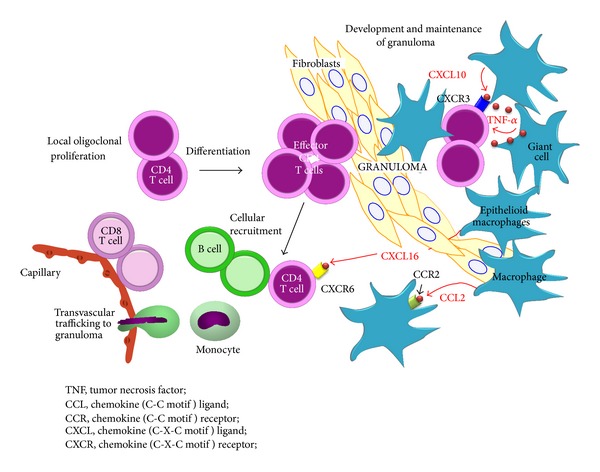
Recruitment of inflammatory cells and granuloma formation. Release of type 1 helper T (Th1) cytokines or chemokines promotes cellular accumulation and nidus formation, resulting in granuloma. Activated macrophages increase the expression of co-stimulatory molecules; released chemokines such as CXCL10 attract additional T cells with a CD4/Th1 phenotype.

The histologic hallmark of sarcoidosis, irrespective of organ involvement, is noncaseating epithelioid granulomas. Granulomas typically consist of a compact central area of macrophages that differentiate into epithelioid cells and then fuse to form multinucleated giant cells surrounded by lymphocytes (Figure 1) [3]. These cells become the primary sources for angiotensin-converting enzyme (ACE). Lymphocytes scattered within granulomas tend to be CD4+ T helper cells, while those around the periphery are CD8+ T cells and to a lesser extent B cells (Figure 1).

There is generally minimal necrosis within sarcoid granulomas, unlike those associated with* Mycobacterium tuberculosis* infection [13]. Granulomas in sarcoidosis are thought to form around and isolate poorly degraded antigens as a means of preventing antigen dissemination and further tissue damage. Why sarcoidosis spontaneously resolves in some patients and progresses in others is still poorly understood. With respect to progressive disease, it has been postulated that the antigen persists, thereby inducing a chronic immune response [14, 15]. Importantly, the detection of granulomas alone is not specific enough to diagnose sarcoidosis, especially noncaseating granulomas that are rarely found by subendomyocardial biopsy in CS [16]. Sarcoidosis is conventionally diagnosed by the appropriate combination of clinical, physiologic, and multimodality imaging. Histological findings of granuloma mainly serve to support, not definitively confirm, the diagnosis of sarcoidosis and exclude other potential etiologies from the differential diagnosis.

## 5. Manifestations

The incidence of CS varies by ethnic group and depends on the type of study performed. Studies based on clinical findings in known sarcoid patients are likely given a prevalence of cardiac involvement in 5% to 10% of affected patients [17]. An excellent American autopsy series published in 1978 showed that 27% of 84 autopsied patients with a diagnosis of sarcoidosis had CS, two-thirds of whom had clinically significant cardiac disease [18]. Although myocardial fibrosis is nonspecific, it can sometimes be the only manifestation of CS. This series did not consider myocardial fibrosis as evidence of cardiac involvement, so it likely underestimated the true prevalence of CS. A recent American study investigated 62 outpatients with documented sarcoidosis and no documented CS. They asked about cardiac symptoms and performed noninvasive tests (simple electrocardiography [ECG], Holter monitoring, and transthoracic echocardiography). Any patient who reported symptoms or had an abnormal study was sent for advanced imaging including cardiac magnetic resonance imaging (MRI) or ^18^F-fluoro-2-deoxyglucose positron emission tomography (^18^F-FDG PET). The results showed that almost 40% of outpatients with documented sarcoid had CS. Of these, slightly more than half were asymptomatic [19]. CS is most common among older, female Japanese sarcoid patients, with a reported rate of cardiac involvement of almost 80% [20].

**Figure 2:**
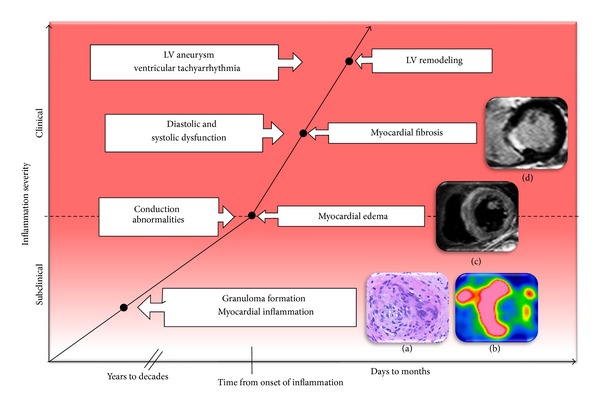
Imaging and inflammation cascade. In this schematic representation of disease progression, the infection point is represented by inflammation, edema, and fibrosis. Granuloma formation (a), ^18^F-FDG PET of myocardial inflammation (b), cardiac magnetic resonance imaging of myocardial edema (c), and cardiac magnetic resonance imaging of myocardial fibrosis (d) are shown. ^18^F-FDG PET: ^18^F-fluorodeoxyglucose positron emission tomography.

CS can affect any part of the heart and its conduction system. The most frequently involved area is the ventricular septum (31.5%), followed by the inferior wall, anterior left ventricle, right ventricle, and lateral left ventricle [21]. Sarcoid granulomas and subsequent fibrosis may induce complete atrioventricular (AV) block, systolic and diastolic dysfunction, and ventricular tachycardia (VT) (Figure 2) [22–27]. Patients may present with syncope, heart failure, or sudden death. Because CS often occurs in the absence of apparent disease elsewhere, sarcoidosis should be considered in any nonischemic form of cardiomyopathy, particularly when rhythm disturbances are prominent.

A precise and early diagnosis would be needed to introduce effective anti-inflammatory therapy that could prevent an adverse outcome [19, 28]. However, the lack of gold standard in the diagnosis of CS remains a major problem.

## 6. Japanese Guidelines for the Diagnosis of CS

Clinical guidelines for the diagnosis of CS were first published by the Japanese Ministry of Health and Welfare (JMHW) in 1993 and they have been used most commonly in the diagnosis of CS and as the reference for comparison of various imaging techniques [29]. Electrophysiological studies should be used to evaluate patients with syncope or significant ECG abnormalities, but their sensitivity and ability to stratify patients by risk are poorly defined. A normal electrophysiological study at any one point does not predict future granulomatous infiltration and fibrosis in critical regions. This means that advanced imaging modalities such as cardiac MRI and ^18^F-FDG PET are needed to assess disease activity and management. In 2006, the Joint Committee of the Japan Society of Sarcoidosis and Other Granulomatous Disorders and the Japanese College of Cardiology published a revised version of the guidelines, in which delayed enhancement (DE) in cardiac MRI was added as a minor criterion for the clinical diagnosis as shown in the following list [30, 31].

Japanese Ministry of Health and Welfare Criteria for Cardiac Sarcoidosis

*Major Criteria*

- Advanced atrioventricular block.
- Basal thinning of the interventricular septum.
- Positive 67 gallium uptake in the heart.
- Depressed ejection fraction of the left ventricle (50%).

*Minor Criteria*

- Abnormal ECG findings: ventricular arrhythmias (ventricular tachycardia, multifocal or frequent PVCs), CRBBB, axis deviation or abnormal Q-wave.
- Abnormal echocardiography: regional abnormal wall motion or morphological abnormality (ventricular aneurysm, wall thickening).
- Nuclear medicine: perfusion defect detected by 201 thallium or 99 m technetium myocardial scintigraphy.
- Gadolinium-enhanced CMR imaging: delayed enhancement of myocardium.
- Endomyocardial biopsy: interstitial fibrosis or monocyte infiltration over moderate grade.
- CMR: cardiac magnetic resonance, CRBBB: complete right bundle branch block, ECG: electrocardiogram, and PVC: premature ventricular contraction.
- Two or more of the four major criteria are satisfied.
- One in two of the major criteria and two or more of the five minor criteria are satisfied.

Abnormal cardiac accumulation on ^18^F-FDG PET was not added to the diagnostic criteria but was included in the additional statements. Several imaging studies have been published on utilizing ^18^F-FDG PET and cardiac MRI for the diagnosis of CS to date, but almost all of them were validated using JMHW's original guidelines [32–40]. Two recent studies did, however, examine the diagnostic accuracy of ^18^F-FDG PET based on the revised guidelines (Table 1) [37, 40].

## 7. Cardiac MRI

Cardiac MRI has emerged as the gold standard for the diagnosis of cardiac involvement in sarcoidosis. Early enhancement of sarcoid granulomas in T2-weighted images suggests the presence of inflammation and edema, whereas DE indicates fibrotic changes and scarring [41, 42]. Commonly, the two phases overlap. Smedema et al. evaluated the diagnostic role of cardiac MRI in 58 patients with suspected CS and reported that DE showed favorable sensitivity and specificity of 100% and 78%, respectively [42]. Kim et al. compared the prognostic value of DE in cardiac MRI with JMHW's criteria in an asymptomatic cohort of 81 patients with biopsy-proven extra-cardiac sarcoidosis [43]. CS was detected in 26% of patients by DE on cardiac MRI, but only 12% fulfilled JMHW's criteria. DE was associated with adverse events and cardiac death. These results were confirmed by a more recent study, in which 155 patients with extra-cardiac sarcoidosis underwent cardiac MRI and had a follow-up of approximately 2.6 years [41]. In addition to reliably, detecting the disease, DE may also be useful for assessing the response to steroid therapy [44, 45]. Unfortunately, however, the test is not effective for patients with non-MRI compatible pacemakers and defibrillators, or those with renal dysfunction.

## 8. ^**18**^F-FDG PET

^18^F-FDG PET is a noninvasive molecular imaging technique that is highly sensitive to metabolically active processes. ^18^F-FDG is taken up by living cells via cell membrane glucose transporters (GLUT) and is phosphorylated intracellularly by a hexokinase into ^18^F-FDG-6-phosphate (^18^F-FDG-6-P). ^18^F-FDG-6-P cannot be metabolized further along the glycolytic pathway and therefore accumulates within cells in direct proportion to their metabolic activity, a phenomenon known as metabolic trapping. In oncology, the uptake of ^18^F-FDG by tumor cells makes PET the gold-standard technique for investigating metastases [46, 47]. The use of this technique for imaging inflammation has also been proposed, partly because ^18^F-FDG has been noted at sites of inflammation during routine ^18^F-FDG PET imaging of cancer patients [48]. The identification of sites of inflammation is related to the glycolytic activity of cells involved in the inflammatory response. In inflammatory cells, especially neutrophils and monocytes/macrophages, cellular activation increases GLUT1 and GLUT3 in the cell membrane and hexokinase over the levels in resting cells [49, 50].

Under aerobic conditions, most myocardial energy consumption is derived from oxidation of free fatty acids (FFAs), followed by glucose and, to a smaller extent, amino acids. In the myocardium with a low concentration of glucose-6-phosphatase, ^18^F-FDG-6-P does not enter into further enzymatic pathway and accumulates intracellularly, proportional to the glycolytic rate of the cell.

## 9. ^**18**^F-FDG PET in the Diagnosis of CS

Recent studies have demonstrated the promising potential of ^18^F-FDG PET in the diagnosis and assessment of CS [32–40, 51–53]. Accumulation of ^18^F-FDG is associated with an active inflammatory process in patients with CS [54, 55]. Gallium 67 (Ga-67) scintigraphy has long been used for the diagnosis of CS, but it is now being replaced by ^18^F-FDG PET, mainly because of its low diagnostic sensitivity, which does not exceed 36% owing to low image resolution [34, 35, 38]; ^18^F-FDG PET has a markedly higher sensitivity (71–100%) for diagnosing CS in comparison (Table 1). Advances continue to be made in PET/CT technology, and ^18^F-FDG PET offers less exposure to radiation, quantitative analysis capability, and greater sensitivity for diagnosing CS than other radioisotope imaging modalities such as thallium-201 or technetium-99 m perfusion single photon emission CT [34, 35, 38]. Langah et al. also reported favorable sensitivity (85%) and specificity (90%) of ^18^F-FDG/CT in patients with suspected CS [36] (Table 1), while a series of case reports have hinted at the promise value of ^18^F-FDG in detecting cardiac sarcoid lesions [56, 57]. For example, Smedema et al. reported that increased ^18^F-FDG uptake indicated active myocardial inflammation in the heart in a patient with biopsy-proven CS [56]. Similarly, Koiwa et al. demonstrated that ^18^F-FDG uptake in the heart corresponded well with the pathologically confirmed sarcoid lesions in an autopsy case [57]. Two studies of the diagnostic accuracy of ^18^F-FDG PET have been published and were based on the JMHW revised guidelines in 2006. Tahara et al. reported that they utilized visual and quantitative analysis using ^18^F-FDG PET images [37]. Mc Ardle et al. reported the sensitivity and specificity for diagnosing CS as 100% and 83%, respectively, and that CS patients with VT displayed higher cardiac uptake of ^18^F-FDG when compared with those with advanced AV block [51]. The specificity of ^18^F-FDG in the detection of CS varies and is relatively low (39–97%) compared with its sensitivity (Table 1). Possible explanations for this include nonspecific myocardial uptake of ^18^F-FDG in the normal heart [58, 59] and early-stage sarcoid lesions in the heart of patients who do not meet the diagnostic criteria for CS [60].

**Figure 3:**
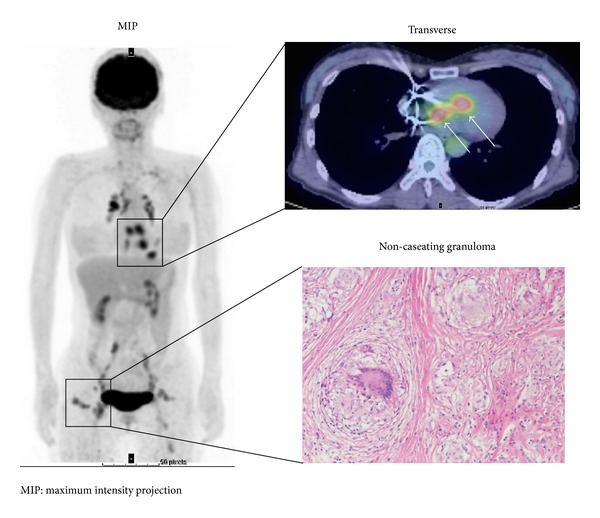
^18^F-FDG PET study in a subject with cardiac and extra-cardiac sarcoidosis. ^18^F-FDG uptake is focally increased in the basal septal wall, consistent with active CS, and is suppressed in other left ventricular walls (white arrows). Whole-body study shows multiple extra-cardiac foci of ^18^F-FDG uptake in chest, lymph nodes, and subcutaneous tissue consistent with active extensive systemic sarcoidosis. ^18^F-FDG PET: ^18^F-fluorodeoxyglucose positron emission tomography, MIP: maximum intensity projection.

Cardiac assessment can be combined with whole-body imaging to determine the presence and activity of extra-cardiac sarcoidosis lesions (Figure 3).

## 10. Minimizing Physiological ^**18**^F-FDG Uptake in the Normal Myocardium

The identification of sites of inflammation is related to the glycolytic activity of the cells involved in the inflammatory response. It is important to determine whether ^18^F-FDG uptake in inflammatory lesions can be distinguished from physiological ^18^F-FDG uptake in the myocardium. Although a focal uptake pattern is most suggestive of CS (Figure 3), several studies have reported that fasting ^18^F-FDG uptake is inhomogeneous throughout the left ventricle in healthy subjects [61] and in oncologic patients [62], with regional maximal uptake in the infero-lateral and basal myocardium, probably because of local differences in substrate use [62]. This physiological ^18^F-FDG uptake by the normal myocardium is problematic because it could lead to blurring of the sarcoid lesions in the heart and/or false-positive results. Although there is currently no consensus on the best protocol for suppressing cardiac ^18^F-FDG uptake, a recently proposed protocol that combines prolonged fasting [63], dietary modifications [64, 65], and unfractionated heparin load before imaging [35] appears to be an attractive solution. In the fasting condition, normal myocardial cells use FFAs for up to 90% of their oxygen consumption [66, 67]. In contrast, when plasma glucose or insulin levels are increased after eating, glucose use may become dominant over FFA use. For clear visualization of CS lesions with a high signal-to-noise ratio, fasting imaging is preferable to postprandial imaging because background ^18^F-FDG uptake is more suppressed [68]. Williams and Kolodny have shown that a very high-fat, low-carbohydrate, protein-permitted diet suppresses myocardial ^18^F-FDG uptake more effectively than overnight or 4 h fasting in an oncologic cohort [65]. Comparing the effectiveness of myocardial ^18^F-FDG suppression with a low-carbohydrate, high-fat, and protein-permitted diet with prolonged fasting over 12 h in an oncologic cohort, Harisankar et al. found that dietary restriction better suppressed cardiac uptake than did prolonged fasting [64]. Unfractionated heparin increases plasma FFAs levels via activation of lipoprotein and hepatic lipases [69], which may cause a reduction of glucose consumption in the normal myocardium. In a Japanese CS study cohort after a fasting period of at least 6 h, 50 units of unfractionated heparin per kilogram of body weight were injected 15 min before application of ^18^F-FDG [35]. This resulted in robust suppression of cardiac ^18^F-FDG uptake. However, a more recent study comparing heparin with prolonged fasting of more than 17.5 h reported that cardiac uptake was inhibited to a greater degree by extended fasting [70].

If there is a more diffuse uptake, the suppression of myocardial physiologic uptake may have been insufficient and other pitfalls should be considered. These include insufficient myocardial uptake suppression leading to heterogeneous ^18^F-FDG uptake (maximum in the basal and lateral walls); other nonischemic cardiomyopathy or other inflammatory diseases of the heart leading to a substrate shift toward glucose, resulting in (heterogeneous) myocardial ^18^F-FDG uptake; and myocardial ischemia resulting in sustained regionally increased myocardial ^18^F-FDG uptake. Maurer et al. have shown that total cardiac uptake activity was suppressed in only 9% of an oncologic patient cohort despite adequate fasting [62]. Additionally, Israel et al. demonstrated that cardiac ^18^F-FDG uptake was significantly higher in male patients, patients younger than 30 years, patients who had fasted for less than 5 h, patients with heart failure, and patients receiving benzodiazepines [71].

## 11. Analysis of ^**18**^F-FDG PET Image

### 11.1. Semiquantitative Analysis

Focal myocardial ^18^F-FDG uptake has been considered a finding suggestive of active CS lesions. Ishimaru et al. visually classified ^18^F-FDG PET uptake into four patterns—“none,” “diffuse,” “focal,” and “focal on diffuse”—where the latter two patterns were considered to be indicative of CS [32, 35]. Similarly, Langah et al. defined the patterns of ^18^F-FDG uptake in the heart as “diffuse” or “focal” [36]. Alternatively, Yamagishi et al. divided the left ventricle into nine segments and defined a normal perfusion segment on cardiac ^13^N-NH3 images as the control region. They then compared ^18^F-FDG uptake between the segments with increased ^18^F-FDG uptake and the control segment [38]. ^18^F-FDG PET is often combined with a perfusion scan and ECG gating to rule out coronary artery disease or to identify resting perfusion defects suggestive of inflammation-induced tissue damage [51]. Normal perfusion and increased focal ^18^F-FDG uptake represent early CS, whereas abnormal perfusion and increased ^18^F-FDG uptake more likely represent advanced disease with tissue damage. Scarring, a potential outcome of end-stage disease, may result in abnormal perfusion without ^18^F-FDG uptake [34]. In regard to segmental analysis, Ohira et al. and Yamagishi et al. used 16-segment and 9-segment models of the left ventricle, respectively (Table 1).

### 11.2. Quantitative Analysis

The standardized uptake value (SUV) is commonly used as an index of tracer uptake in tumor imaging. The SUV can be obtained using the following equation. SUV = (decay-corrected radiotracer concentration, mCi/mL)/(mCi of tracer injected dose into the patient)(body weight). In CS, a high SUV in the ^18^F-FDG uptake site has been reported, consistent with visual determination of the site [34]. Okumura et al. divided the left ventricular cardiac muscle into 13 segments. Patients with CS showed a higher myocardial SUV than did healthy subjects. The diagnostic capability based on the above criterion had a sensitivity of 100% and specificity of 91% [34]. Tahara et al. reported that they utilized visual analysis using ^18^F-FDG PET images, and the sensitivity and specificity for diagnosing CS were 100% and 46%, respectively, but after the quantitative analysis utilizing the coefficient of variation of SUV for segmental ^18^F-FDG uptake, they achieved a specificity of 97% (Table 1) [37]. As for the use of SUV in the diagnosis of CS, problems that remain to be solved included unstandardized SUV calculation software and differences in measurement values depending on imaging equipment and imaging conditions.

## 12. Assessment of Disease Activity during Corticosteroid Treatment

**Figure 4:**
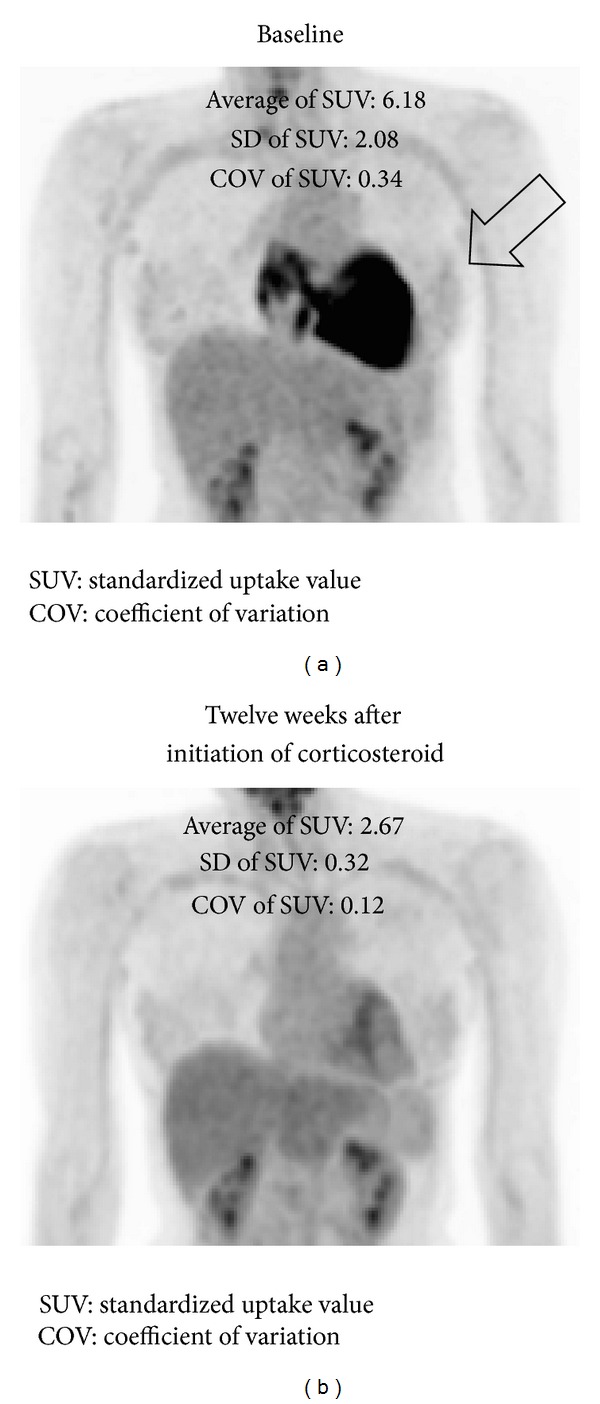
^18^F-FDG PET before and after corticosteroid therapy. (a) ^18^F-FDG PET image (3D maximum intensity projection) before therapy. (b) After administration of 30 mg/day prednisone for 12 weeks, FDG uptake (arrow) has almost disappeared. ^18^F-FDG PET: ^18^F-fluoro-2-deoxyglucose positron emission tomography. SUV: standardized uptake value, COV: coefficient of variation.

The metabolic signal of inflammation is a marker of disease activity and can be used to guide the need for and response to corticosteroid therapies (Figure 4) [37, 38, 53, 72].

Among the case reports in the literature, Takeda et al. reported a case of CS with third-degree AV block that had markedly increased ^18^F-FDG uptake in the basal interventricular septum. After corticosteroid therapy, myocardial ^18^F-FDG uptake disappeared and the complete AV block became a first-degree AV block [53]. Similarly, Tadamura et al. documented a case of reduced regional ^18^F-FDG uptake in the heart after corticosteroid therapy. In their case, myocardial ^18^F-FDG uptake decreased, as did the serum level of ACE, a biomarker that reflects the disease activity of sarcoidosis [72]. After steroid therapy, a small study in 17 biopsy-proven CS patients showed a significant decrease in ^18^F-FDG uptake, while perfusion defects remained stable [38]. Three patients showed improvement on ECG as well. Pandya et al. reported that the recurrence of symptomatic VT was predicted by increased ^18^F-FDG uptake during corticosteroid tapering [73]. In a recent prospective study of 28 patients with biopsy-proven sarcoidosis, ^18^F-FDG PET/CT influenced clinical management in 63% [63]. These reports suggest the practical role of ^18^F-FDG PET in monitoring the disease activity of CS during corticosteroid therapy; however, there are pitfalls in this. First, steroids often induce glucose intolerance along with gluconeogenesis and insulin resistance. Serum glucose and insulin levels elevated by steroid therapy could affect ^18^F-FDG uptake in target organs including the heart [74, 75]. This may preclude an accurate assessment of the effects of corticosteroid using ^18^F-FDG PET. Also, ^18^F-FDG PET has a limited ability in showing fibrous regions, although corticosteroid therapy may facilitate the transition from active inflammatory changes to the fibrotic stage. Fibrous lesions can be a focus of reentrant VT. Therefore, decreased ^18^F-FDG uptake in the heart should not necessarily be interpreted as a favorable finding.

## 13. Biomarkers of CS

There are several parameters that can be used to monitor the inflammation of sarcoidosis, including serum ACE, lysozyme, and soluble IL-2 receptor [76–78]. Although ACE is a clinically useful biochemical marker of systemic sarcoidosis, serum levels soon return to the normal range after corticosteroid therapy even when disease activity remains high [79]. ACE inhibitors are often used to treat heart failure in CS, and serum ACE levels are therefore likely to be influenced by this treatment.

## 14. Comparing ^**18**^F-FDG PET and Cardiac MRI

**Figure 5:**
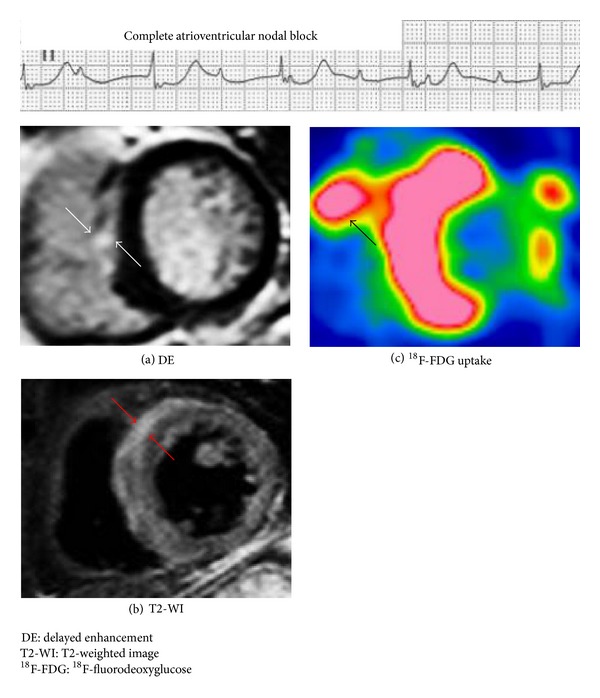
Representative images of cardiac MRI and ^18^F-FDG PET in a CS patient with complete atrioventricular nodal block. Images of ^18^F-FDG PET and cardiac MRI in a 66-year-old woman with pathologically proven CS. (a), (b) Cardiac MRI shows areas of DE and increased T2-weighted signal in the anteroseptal wall of the left ventricle (white and red arrows). (c) ^18^F-FDG PET shows focal ^18^F-FDG uptake in the anteroseptal of the left ventricle and right ventricle (black arrow). When compared with the ^18^F-FDG PET image, the distribution of the positive finding on MRI notably differs. CS: cardiac sarcoidosis, DE: delayed enhancement, MRI: magnetic resonance imaging, T2-WI: T2-weighted image, ^18^F-FDG PET: ^18^F-fluorodeoxyglucose positron emission tomography.

^18^F-FDG PET detects active CS lesions, whereas DE in cardiac MRI primarily detects more advanced fibrotic lesions [32, 35]. Commonly, the two phases overlap, so the mild to moderate correlation between DE in cardiac MRI and ^18^F-FDG PET is not surprising (Figure 5) [32, 80]. Several studies compared DE in cardiac MRI with ^18^F-FDG uptake in PET. In a report by Mehta et al., ^18^F-FDG showed positive findings in 86% of 22 patients with suspected CS, whereas DE in cardiac MRI showed them in only 36% of patients [19]. Ohira et al. similarly reported that ^18^F-FDG PET (87.5%) was more sensitive than DE in cardiac MRI (75%) [32]. In their study, however, the specificity of ^18^F-FDG PET (39%) was obviously lower than that of DE in cardiac MRI (88%). When DE in cardiac MRI and ^18^F-FDG PET were compared against JMHW's criteria, DE in cardiac MRI had a higher specificity but a lower sensitivity [19, 32, 81].

Cardiac MRI has greater spatial and temporal resolution compared with ^18^F-FDG PET without radiation exposure. It also allows for anatomical and hemodynamic assessment of cardiac function. However, cardiac MRI is not feasible in patients with implantable devices in the heart, which are often used for CS.

The advantages of ^18^F-FDG PET include the biologic nature of the imaging signal, the potential for identifying cardiac and extra-cardiac sarcoidosis involvement (Figure 3) [40], and the feasibility of imaging in patients with implantable cardiac devices. Another advantage for therapy or risk stratification may be quantification. According to the European Association of Nuclear Medicine (EANM) and Society of Nuclear Medicine and Molecular Imaging guidelines for ^18^F-FDG in inflammation, SUV should be used with caution in this setting [48]. However, McArdle et al. found higher quantitative ^18^F-FDG uptake in CS patients with VT than in those with AV block and asymptomatic controls [40]. Similarly, Blankstein et al. concluded after examining a group of 125 patients that abnormal cardiac PET findings, but not JMHW criteria or the ejection fraction, are associated with a higher risk of death or VT [33].

The disadvantages of ^18^F-FDG PET include radiation exposure, false-positive result of cardiac uptake, and the inability to detect smaller regions of myocardial damage. Also, a potential source of error in patients with an implantable cardiac device may be hot-spot artifacts at the lead insertion site on attenuation-corrected images. Although some studies have suggested a significant overestimation of SUV [82], others have suggested that images in the presence of metallic leads can be interpreted without correction for metal artifacts [83]. Evaluation of images without attenuation may be used as an adjunct in cases of suspected lead insertion artifacts.

## 15. Future Directions

**Figure 6:**
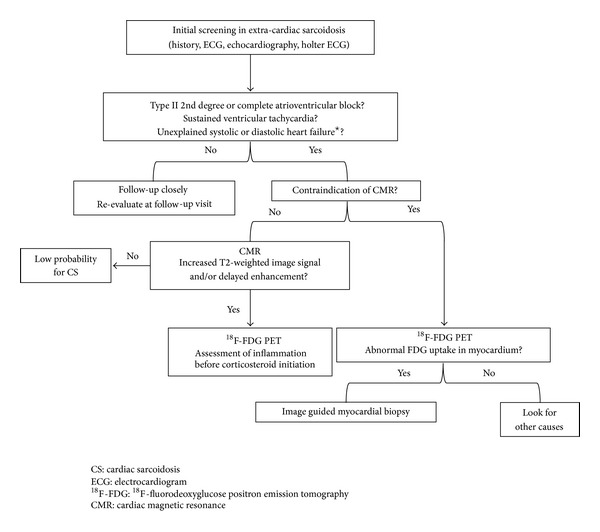
Diagnostic algorithm for a patient with suspected for cardiac involvement in extra-cardiac sarcoidosis. ^★^Absence of coronary artery disease as determined by selective coronary angiography and the absence of a comorbidity that could explain heart failure. Patients with initially negative screening tests should have repeat evaluations at follow-up visits. Cardiac MRI is the most specific test and ^18^F-FDG PET is the most sensitive test available for cardiac sarcoidosis. ^18^F-FDG PET should be considered for patients with positive findings on cardiac MRI to assess inflammatory activity before initiating corticosteroid therapy. ^18^F-FDG PET-guided myocardial biopsy should be performed in patients with a contraindication to cardiac MRI.

The goals for management of patients with CS are to preserve cardiac function and avoid fatal arrhythmia, thereby achieving better quality and longer survival. Advanced imaging such as cardiac MRI and ^18^F-FDG-PET may be useful for highly accurate early diagnosis, assessment of inflammatory activity, and therapeutic monitoring. Moreover, if optimal risk stratification of CS becomes available in the clinical setting, better prognosis is likely to be achieved in the future. However, a stepwise approach to diagnosing patients with cardiac involvement has not been established. Initial screening in extra-cardiac sarcoidosis usually includes a detailed medical history and physical exam, a surface ECG, and a transthoracic echocardiogram (Figure 6). Significant ECG abnormality such as new AV block or VT and echocardiographic abnormality such as decreased systolic/diastolic LV function should prompt further evaluation, usually with Holter monitoring and additional cardiac imaging [84]. Patients with initially negative screening tests should have repeat evaluations at follow-up visits to improve the sensitivity for detecting cardiac involvement [20]. In patients with initially positive screening tests, cardiac MRI should be performed because of its high specificity. Accordingly, ^18^F-FDG PET should be considered in patients with positive findings on cardiac MRI to assess inflammatory activity before initiating corticosteroid therapy. On the other hand, ^18^F-FDG PET-guided myocardial biopsy should be performed in patients with a contraindication to cardiac MRI (Figure 6).

**Figure 7:**
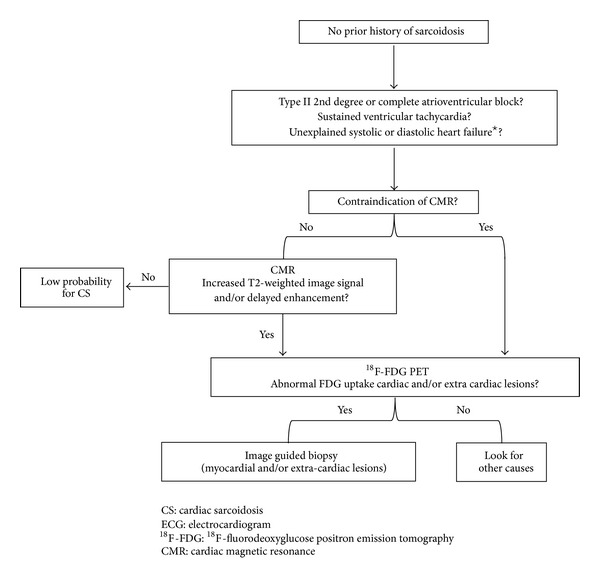
Diagnostic algorithm for a patient with suspected for cardiac sarcoidosis. ^★^Absence of coronary artery disease as determined by selective coronary angiography and the absence of a comorbidity that could explain heart failure. Cardiac MRI is the most specific test and ^18^F-FDG PET is the most sensitive test available for cardiac sarcoidosis. In patients without a prior history of sarcoidosis, cardiac MRI should be considered if they have type II 2nd degree or complete atrioventricular block, sustained ventricular tachycardia, or unexplained systolic or diastolic LV dysfunction. ^18^F-FDG PET should be performed in patients with positive findings on cardiac MRI or contraindication to cardiac MRI to detect both cardiac and extra-cardiac sarcoid lesions, and ^18^F-FDG PET-guided biopsy should be performed.

In patients without a prior history of sarcoidosis, cardiac MRI should be considered if they have type II 2nd degree or complete AV block, sustained VT, or unexplained systolic or diastolic LV dysfunction. ^18^F-FDG PET should be performed in patients with positive findings on cardiac MRI or contraindication to cardiac MRI to detect both cardiac and extra-cardiac sarcoid lesions, and they should undergo ^18^F-FDG PET-guided biopsy (Figure 7). Clinical benefits and the cost effectiveness of these approaches should be evaluated simultaneously.

In the last decade, ^18^F-FDG PET has substantially enhanced the detection of CS. However, no noteworthy progress has been made in the treatment of CS, and the prognosis of patients with CS has not notably improved. This means that there are still some problems to be worked out in the use of ^18^F-FDG PET. First, standardized preparation protocols should be established to suppress physiological ^18^F-FDG uptake sufficiently in the normal myocardium. This will likely help detect early-stage sarcoid lesions with a favorable sensitivity and specificity. Several protocols have already been introduced, but more easily applicable and reliable preparations are needed. Recently, dual time-point ^18^F-FDG PET delayed imaging has been reported to be useful, not only for diagnosing malignancies [85], but also for assessing the activity of benign inflammatory disease [86]. In stimulated inflammatory cells, hexokinase, which mediates the phosphorylation of intracellular ^18^F-FDG and results in its retention within cells, is translocated to the endofacial surface of the glucose transporter and is activated [87, 88]. Therefore, activation of inflammatory cells causes a sustained increase in the accumulation of ^18^F-FDG over time. In a pulmonary sarcoidosis study, dual time-point ^18^F-FDG PET was useful over a 1-year observation period for detecting patients with persistent pulmonary disease [89]. However, there has been no data reported on dual time-point ^18^F-FDG PET delayed imaging in CS. Further studies needed to investigate how we can distinguish inflammatory lesions from physiological ^18^F-FDG uptake in the myocardium using this method. Second, a standardized methodology that enables quantitative and objective image analysis should be established. The Japanese Society of Nuclear Cardiology has published “Recommendations standardized for ^18^F-FDG PET imaging for CS” and stated that measurement of SUV in image interpretation may be useful for improving diagnostic capability and quantitative disease activity [90]. Furthermore, adopting a standardized segmentation and nomenclature system of the heart would allow comparison of ^18^F-FDG PET findings from different institutions [91]. In 2006, EANM launched EANM Research Ltd. (EARL) as an initiative to promote multicenter nuclear medicine and research. In January 2010, EANM published the FDG PET and PET/CT: EANM procedure guidelines for tumour PET imaging: version 1.0 [92] in which quantification of ^18^F-FDG PET is defined as quantification using SUV. The use of SUV in multicenter oncology PET studies requires an inter-institution calibration procedure to facilitate the exchangeability of SUVs between institutions. It is important that all participating institutions use a similar methodology To ensure the exchangeability of SUVs, a minimum set of quality-control procedures is recommended; for example, daily quality control, calibration/cross-calibration of PET or PET/CT camera with the institution's own dose calibrator or against another dose calibrator, inter-institution cross-calibration, and determining “activity recovery coefficients.” Accurate, reproducible, and quantitative assessment using SUV could be obtained by standardizing the methodology. Further studies are needed to apply the methodology to CS. Third, long-term prospective clinical studies are needed to determine the value of imaging for therapy monitoring and risk stratification. Finally, the use of ^18^F-FDG PET combined with other imaging modalities is warranted. Hybrid PET/MRI systems have been recently introduced to medical imaging. In the imaging of CS, several case reports have demonstrated the potential of integrated PET/MRI [93, 94]. Cardiac MRI has been shown to provide good diagnostic performance in CS. However, T2-weighted images, which are particularly important for showing inflammatory activity in CS, are prone to artifacts and often do not yield a consistent image quality, especially in patients with arrhythmia and other motion artifacts [95]. In contrast, ^18^F-FDG PET is useful for assessing inflammatory myocardial disease activity for therapy response evaluation [95]. PET/MRI might emerge as an important modality for the diagnosis of CS and monitoring of its disease activity. Additionally, its lower radiation dose compared with PET/CT will be particularly valuable in the imaging of young patients with potentially curable disease [96].

## Figures and Tables

**Table 1:** Sensitivity and specificity of ^18^F-FDG PET in the diagnosis of CS.

Authors	Year	Subjects studied	JMHW guidelines	No. of patients (*n*)	Fasting time (h)	Sensitivity (%)	Specificity (%)	Comments
Yamagishi et al. [38]	2003	With CS	1993	17	>5	82	NA	First systemic research
Okumura et al. [34]	2004	With sarcoidosis	1993	22	>12	100	91	PET is more sensitive than ^67^Ga scintigraphy
Ishimaru et al. [35]	2005	With sarcoidosis	1993	32	>6	100	82	Pre-administered heparin
Ohira et al. [32]	2008	With suspected CS	1993	21	>12	88	39	Comparing ^18^F-FDG PET and MRI
Langah et al. [36]	2009	With suspected CS	1993	76	>18	85	90	PET CT with prolonged fasting >18 h
Tahara et al. [37]	2010	With suspected CS	2006	24	>12	100	46→97	Analysis using the COV improved specificity
Manabe et al. [39]	2013	With suspected CS	1993	67	>6	96	62	^ 18^F-FDG uptake was related to ECG abnormalities
McArdle et al. [40]	2013	With suspected CS	2006	134	>12	100	83	With a high-fat, low-carbohydrate diet on the day before PET
Blankstein et al. [33]	2013	With suspected CS	1993	118	>3	71	45	With a high-fat, high protein, low-carbohydrate diet

CS: cardiac sarcoidosis, ^18^F-FDG PET: ^18^F-fluorodeoxyglucose positron emission tomography, JMHW: Japanese Ministry of Health and Welfare, COV: coefficient of variation.
